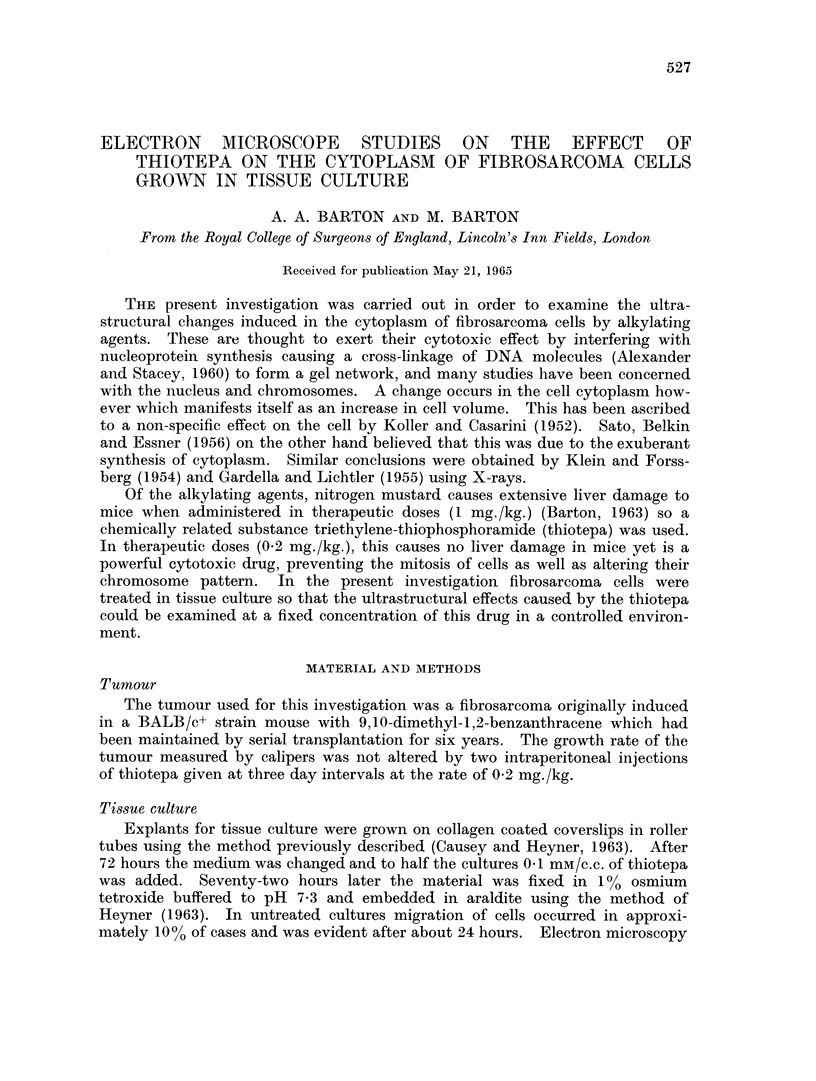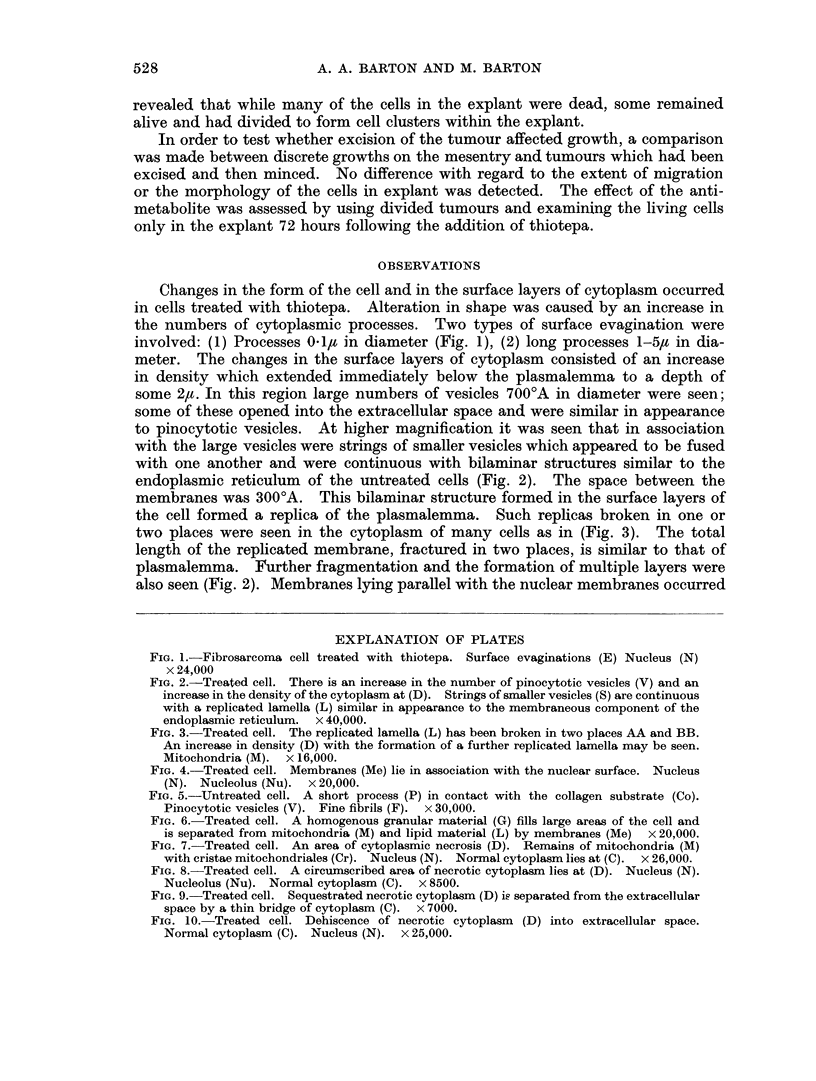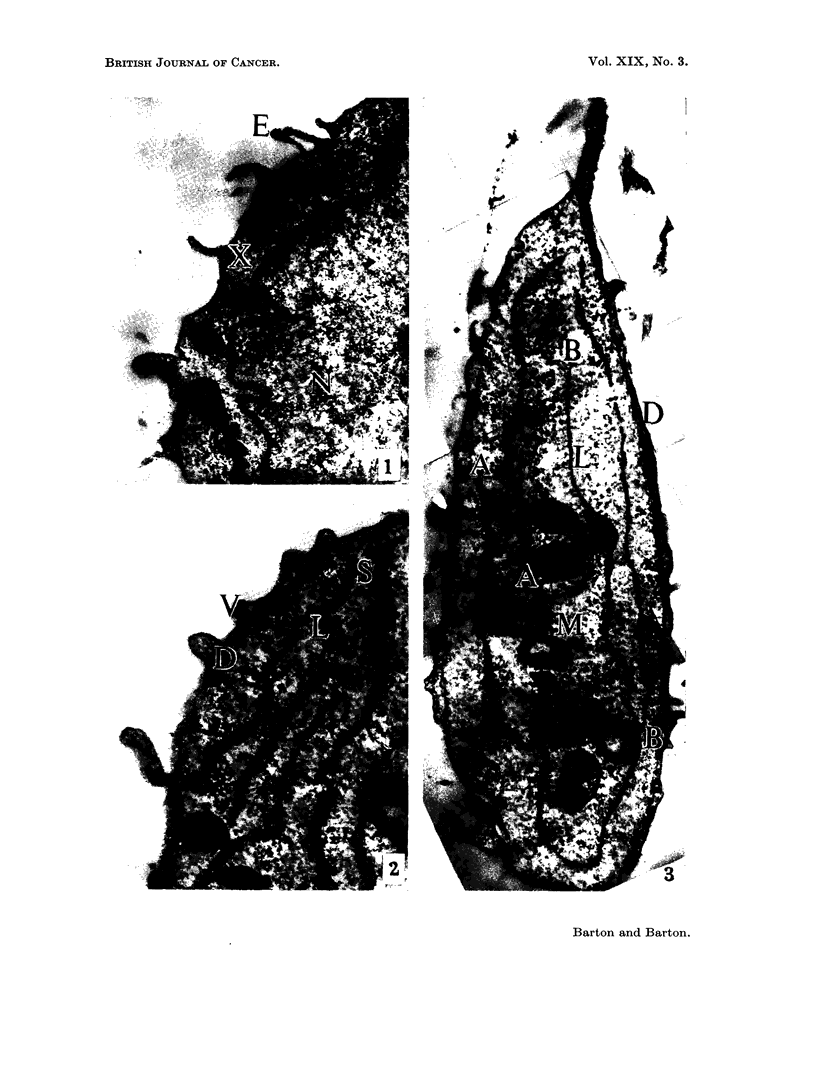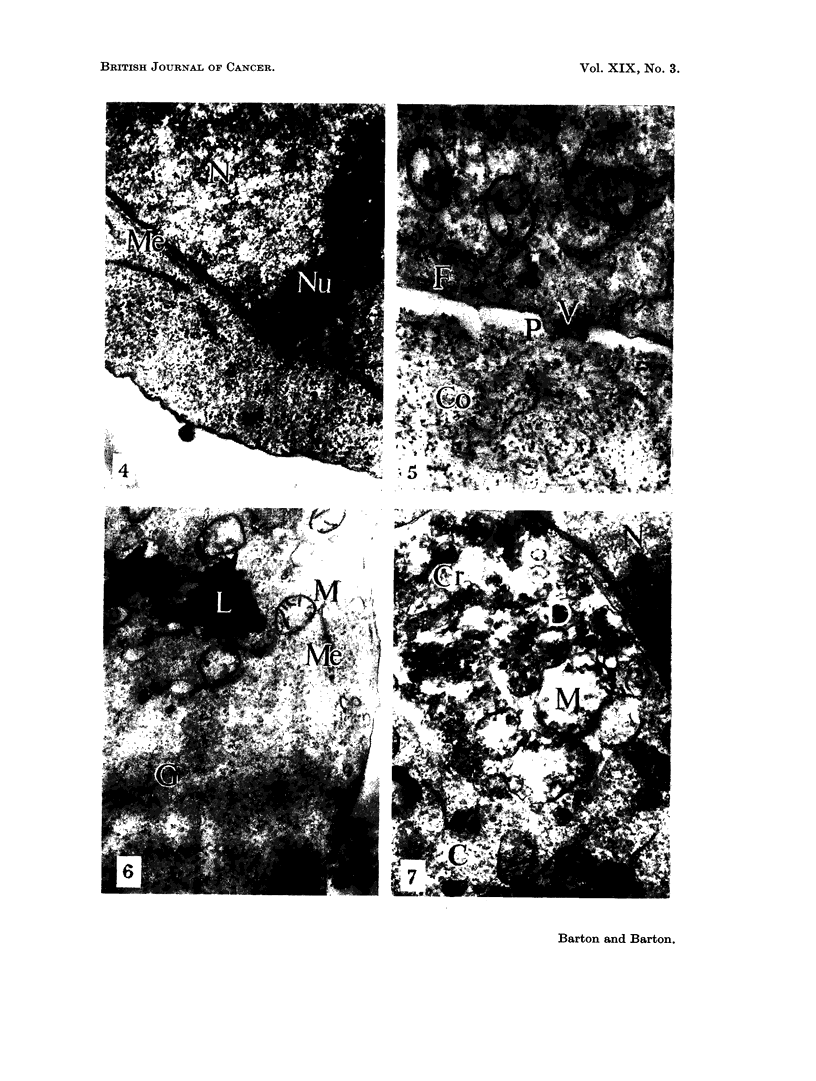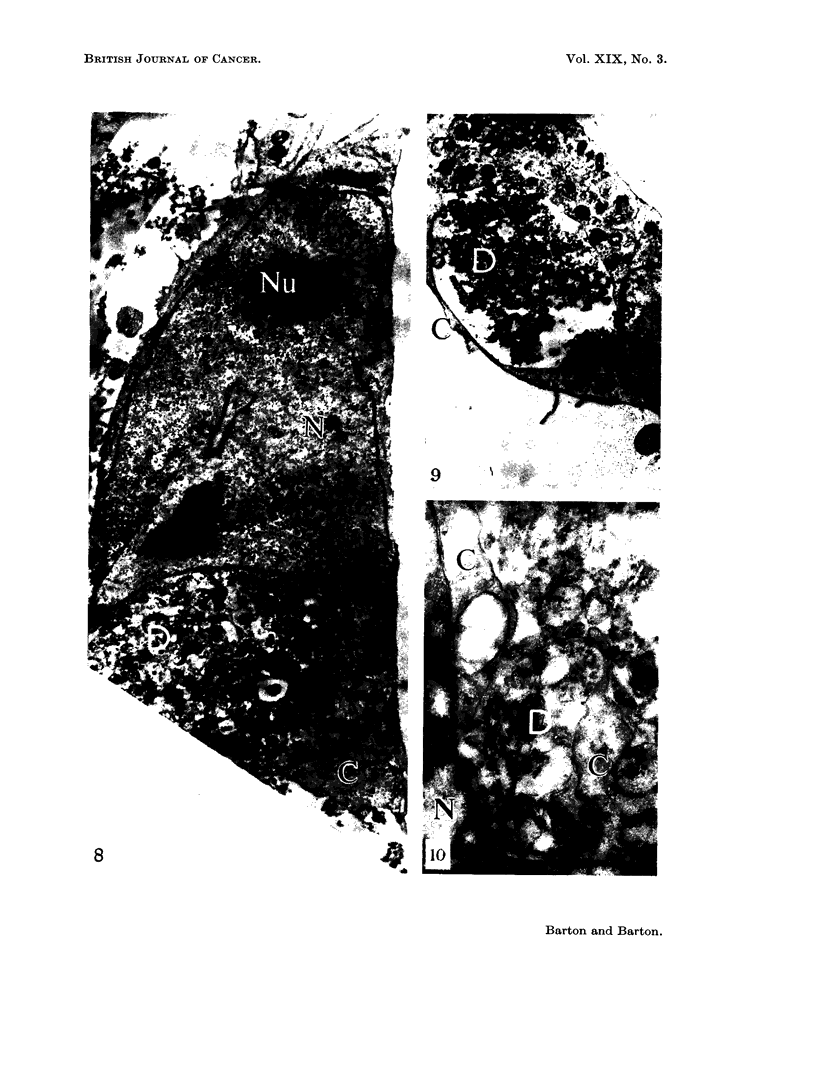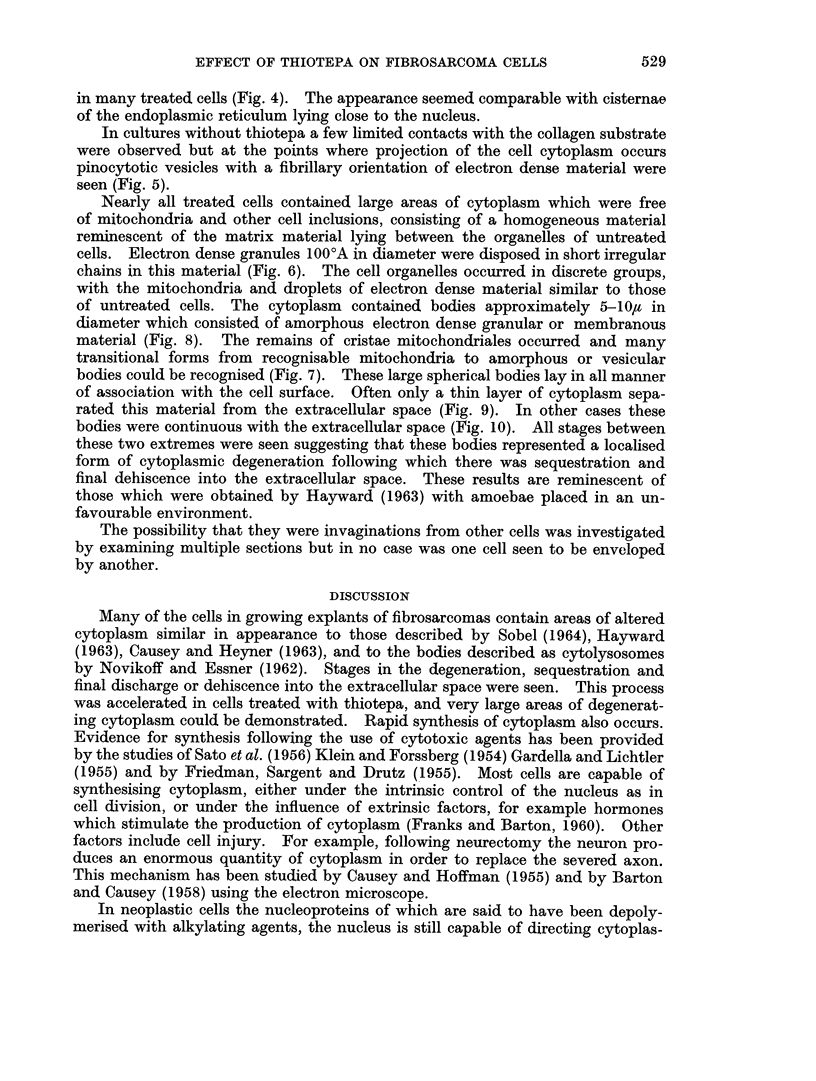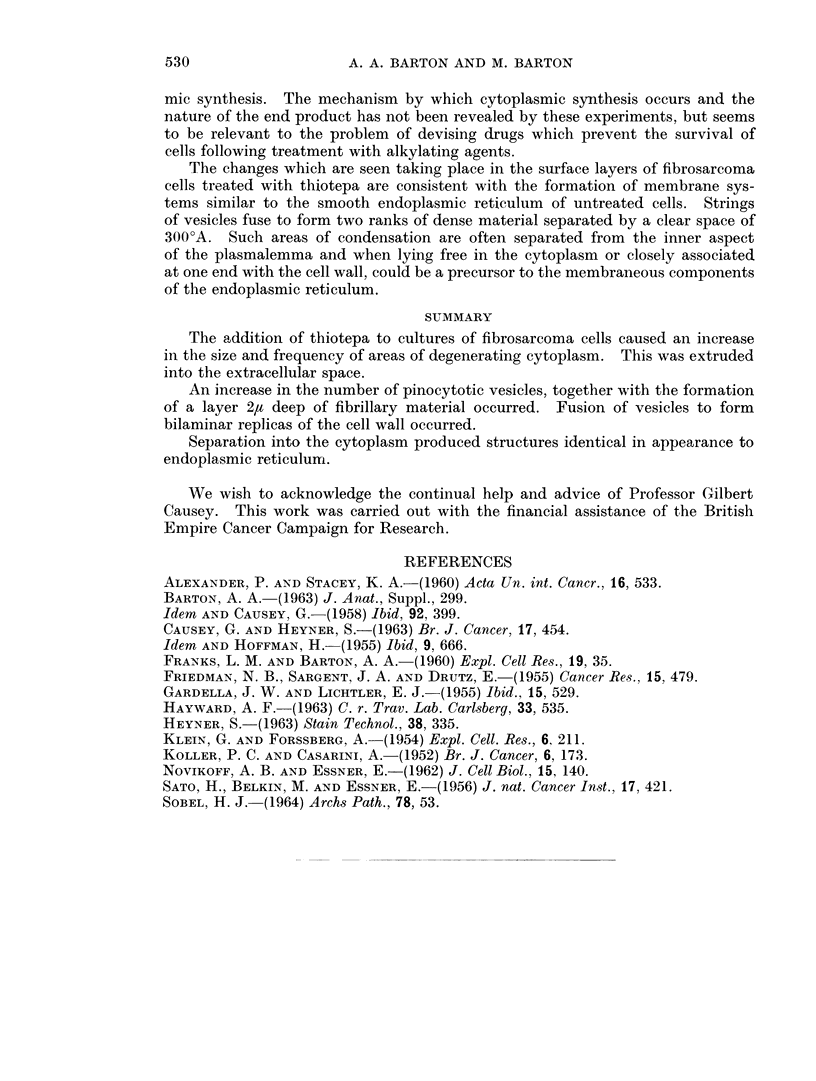# Electron microscope studies on the effect of thiotepa on the cytoplasm of fibrosarcoma cells grown in tissue culture.

**DOI:** 10.1038/bjc.1965.64

**Published:** 1965-09

**Authors:** A. A. Barton, M. Barton

## Abstract

**Images:**


### ELECTRON MICROSCOPE STUDIES ON THE EFFECT OF THIOTEPA ON THE CYTOPLASM OF FIBROSARCOMA CELLS GROWN IN TISSUE CULTURE

A. A. BARTON AND M. BARTON

*From the Royal College of Surgeons of England, Lincoln's Inn Fields, London*

Received for publication May 21, 1965

THE present investigation was carried out in order to examine the ultrastructural changes induced in the cytoplasm of fibrosarcoma cells by alkylating agents. These are thought to exert their cytotoxic effect by interfering with nucleoprotein synthesis causing a cross-linkage of DNA molecules (Alexander and Stacey, 1960) to form a gel network, and many studies have been concerned with the nucleus and chromosomes. A change occurs in the cell cytoplasm however which manifests itself as an increase in cell volume. This has been ascribed to a non-specific effect on the cell by Koller and Casarini (1952). Sato, Belkin and Essner (1956) on the other hand believed that this was due to the exuberant synthesis of cytoplasm. Similar conclusions were obtained by Klein and Forssberg (1954) and Gardella and Lichtler (1955) using X-rays.

Of the alkylating agents, nitrogen mustard causes extensive liver damage to mice when administered in therapeutic doses (1 mg./kg.) (Barton, 1963) so a chemically related substance triethylene-thiophosphoramide (thiotepa) was used. In therapeutic doses (0·2 mg./kg.), this causes no liver damage in mice yet is a powerful cytotoxic drug, preventing the mitosis of cells as well as altering their chromosome pattern. In the present investigation fibrosarcoma cells were treated in tissue culture so that the ultrastructural effects caused by the thiotepa could be examined at a fixed concentration of this drug in a controlled environment.

### MATERIAL AND METHODS

### *Tumour*

The tumour used for this investigation was a fibrosarcoma originally induced in a BALB/$c^+$ strain mouse with 9,10-dimethyl-1,2-benzanthracene which had been maintained by serial transplantation for six years. The growth rate of the tumour measured by calipers was not altered by two intraperitoneal injections of thiotepa given at three day intervals at the rate of 0·2 mg./kg.

### *Tissue culture*

Explants for tissue culture were grown on collagen coated coverslips in roller tubes using the method previously described (Causey and Heyner, 1963). After 72 hours the medium was changed and to half the cultures 0·1 mM/c.c. of thiotepa was added. Seventy-two hours later the material was fixed in 1% osmium tetroxide buffered to pH 7·3 and embedded in araldite using the method of Heyner (1963). In untreated cultures migration of cells occurred in approximately 10% of cases and was evident after about 24 hours. Electron microscopy revealed that while many of the cells in the explant were dead, some remained alive and had divided to form cell clusters within the explant.

In order to test whether excision of the tumour affected growth, a comparison was made between discrete growths on the mesentry and tumours which had been excised and then minced. No difference with regard to the extent of migration or the morphology of the cells in explant was detected. The effect of the anti-metabolite was assessed by using divided tumours and examining the living cells only in the explant 72 hours following the addition of thiotepa.

### OBSERVATIONS

Changes in the form of the cell and in the surface layers of cytoplasm occurred in cells treated with thiotepa. Alteration in shape was caused by an increase in the numbers of cytoplasmic processes. Two types of surface evagination were involved: (1) Processes $0{\cdot}1\mu$ in diameter (Fig. 1), (2) long processes $1$–$5\mu$ in diameter. The changes in the surface layers of cytoplasm consisted of an increase in density which extended immediately below the plasmalemma to a depth of some $2\mu$. In this region large numbers of vesicles 700°A in diameter were seen; some of these opened into the extracellular space and were similar in appearance to pinocytotic vesicles. At higher magnification it was seen that in association with the large vesicles were strings of smaller vesicles which appeared to be fused with one another and were continuous with bilaminar structures similar to the endoplasmic reticulum of the untreated cells (Fig. 2). The space between the membranes was 300°A. This bilaminar structure formed in the surface layers of the cell formed a replica of the plasmalemma. Such replicas broken in one or two places were seen in the cytoplasm of many cells as in (Fig. 3). The total length of the replicated membrane, fractured in two places, is similar to that of plasmalemma. Further fragmentation and the formation of multiple layers were also seen (Fig. 2). Membranes lying parallel with the nuclear membranes occurred

---

### EXPLANATION OF PLATES

FIG. 1.—Fibrosarcoma cell treated with thiotepa. Surface evaginations (E) Nucleus (N) ×24,000

FIG. 2.—Treated cell. There is an increase in the number of pinocytotic vesicles (V) and an increase in the density of the cytoplasm at (D). Strings of smaller vesicles (S) are continuous with a replicated lamella (L) similar in appearance to the membraneous component of the endoplasmic reticulum. ×40,000.

FIG. 3.—Treated cell. The replicated lamella (L) has been broken in two places AA and BB. An increase in density (D) with the formation of a further replicated lamella may be seen. Mitochondria (M). ×16,000.

FIG. 4.—Treated cell. Membranes (Me) lie in association with the nuclear surface. Nucleus (N). Nucleolus (Nu). ×20,000.

FIG. 5.—Untreated cell. A short process (P) in contact with the collagen substrate (Co). Pinocytotic vesicles (V). Fine fibrils (F). ×30,000.

FIG. 6.—Treated cell. A homogenous granular material (G) fills large areas of the cell and is separated from mitochondria (M) and lipid material (L) by membranes (Me) ×20,000.

FIG. 7.—Treated cell. An area of cytoplasmic necrosis (D). Remains of mitochondria (M) with cristae mitochondriales (Cr). Nucleus (N). Normal cytoplasm lies at (C). ×26,000.

FIG. 8.—Treated cell. A circumscribed area of necrotic cytoplasm lies at (D). Nucleus (N). Nucleolus (Nu). Normal cytoplasm (C). ×8500.

FIG. 9.—Treated cell. Sequestrated necrotic cytoplasm (D) is separated from the extracellular space by a thin bridge of cytoplasm (C). ×7000.

FIG. 10.—Treated cell. Dehiscence of necrotic cytoplasm (D) into extracellular space. Normal cytoplasm (C). Nucleus (N). ×25,000.

E
X
N
1

S
V
L
D
2

B
D
A
L
A
M
B
3

Barton and Barton.

Barton and Barton.

in many treated cells (Fig. 4). The appearance seemed comparable with cisternae of the endoplasmic reticulum lying close to the nucleus.

In cultures without thiotepa a few limited contacts with the collagen substrate were observed but at the points where projection of the cell cytoplasm occurs pinocytotic vesicles with a fibrillary orientation of electron dense material were seen (Fig. 5).

Nearly all treated cells contained large areas of cytoplasm which were free of mitochondria and other cell inclusions, consisting of a homogeneous material reminescent of the matrix material lying between the organelles of untreated cells. Electron dense granules 100°A in diameter were disposed in short irregular chains in this material (Fig. 6). The cell organelles occurred in discrete groups, with the mitochondria and droplets of electron dense material similar to those of untreated cells. The cytoplasm contained bodies approximately 5–10$\mu$ in diameter which consisted of amorphous electron dense granular or membranous material (Fig. 8). The remains of cristae mitochondriales occurred and many transitional forms from recognisable mitochondria to amorphous or vesicular bodies could be recognised (Fig. 7). These large spherical bodies lay in all manner of association with the cell surface. Often only a thin layer of cytoplasm separated this material from the extracellular space (Fig. 9). In other cases these bodies were continuous with the extracellular space (Fig. 10). All stages between these two extremes were seen suggesting that these bodies represented a localised form of cytoplasmic degeneration following which there was sequestration and final dehiscence into the extracellular space. These results are reminescent of those which were obtained by Hayward (1963) with amoebae placed in an unfavourable environment.

The possibility that they were invaginations from other cells was investigated by examining multiple sections but in no case was one cell seen to be enveloped by another.

### DISCUSSION

Many of the cells in growing explants of fibrosarcomas contain areas of altered cytoplasm similar in appearance to those described by Sobel (1964), Hayward (1963), Causey and Heyner (1963), and to the bodies described as cytolysosomes by Novikoff and Essner (1962). Stages in the degeneration, sequestration and final discharge or dehiscence into the extracellular space were seen. This process was accelerated in cells treated with thiotepa, and very large areas of degenerating cytoplasm could be demonstrated. Rapid synthesis of cytoplasm also occurs. Evidence for synthesis following the use of cytotoxic agents has been provided by the studies of Sato *et al.* (1956) Klein and Forssberg (1954) Gardella and Lichtler (1955) and by Friedman, Sargent and Drutz (1955). Most cells are capable of synthesising cytoplasm, either under the intrinsic control of the nucleus as in cell division, or under the influence of extrinsic factors, for example hormones which stimulate the production of cytoplasm (Franks and Barton, 1960). Other factors include cell injury. For example, following neurectomy the neuron produces an enormous quantity of cytoplasm in order to replace the severed axon. This mechanism has been studied by Causey and Hoffman (1955) and by Barton and Causey (1958) using the electron microscope.

In neoplastic cells the nucleoproteins of which are said to have been depolymerised with alkylating agents, the nucleus is still capable of directing cytoplasmic synthesis. The mechanism by which cytoplasmic synthesis occurs and the nature of the end product has not been revealed by these experiments, but seems to be relevant to the problem of devising drugs which prevent the survival of cells following treatment with alkylating agents.

The changes which are seen taking place in the surface layers of fibrosarcoma cells treated with thiotepa are consistent with the formation of membrane systems similar to the smooth endoplasmic reticulum of untreated cells. Strings of vesicles fuse to form two ranks of dense material separated by a clear space of 300°A. Such areas of condensation are often separated from the inner aspect of the plasmalemma and when lying free in the cytoplasm or closely associated at one end with the cell wall, could be a precursor to the membraneous components of the endoplasmic reticulum.

### SUMMARY

The addition of thiotepa to cultures of fibrosarcoma cells caused an increase in the size and frequency of areas of degenerating cytoplasm. This was extruded into the extracellular space.

An increase in the number of pinocytotic vesicles, together with the formation of a layer $2\mu$ deep of fibrillary material occurred. Fusion of vesicles to form bilaminar replicas of the cell wall occurred.

Separation into the cytoplasm produced structures identical in appearance to endoplasmic reticulum.

We wish to acknowledge the continual help and advice of Professor Gilbert Causey. This work was carried out with the financial assistance of the British Empire Cancer Campaign for Research.

### REFERENCES

Alexander, P. and Stacey, K. A.—(1960) *Acta Un. int. Cancr.*, **16**, 533.
Barton, A. A.—(1963) *J. Anat.*, Suppl., 299.
*Idem* and Causey, G.—(1958) *Ibid*, **92**, 399.
Causey, G. and Heyner, S.—(1963) *Br. J. Cancer*, **17**, 454.
*Idem* and Hoffman, H.—(1955) *Ibid*, **9**, 666.
Franks, L. M. and Barton, A. A.—(1960) *Expl. Cell Res.*, **19**, 35.
Friedman, N. B., Sargent, J. A. and Drutz, E.—(1955) *Cancer Res.*, **15**, 479.
Gardella, J. W. and Lichtler, E. J.—(1955) *Ibid.*, **15**, 529.
Hayward, A. F.—(1963) *C. r. Trav. Lab. Carlsberg*, **33**, 535.
Heyner, S.—(1963) *Stain Technol.*, **38**, 335.
Klein, G. and Forssberg, A.—(1954) *Expl. Cell. Res.*, **6**, 211.
Koller, P. C. and Casarini, A.—(1952) *Br. J. Cancer*, **6**, 173.
Novikoff, A. B. and Essner, E.—(1962) *J. Cell Biol.*, **15**, 140.
Sato, H., Belkin, M. and Essner, E.—(1956) *J. nat. Cancer Inst.*, **17**, 421.
Sobel, H. J.—(1964) *Archs Path.*, **78**, 53.